# Polymeric nanoparticles (PNPs) for oral delivery of insulin

**DOI:** 10.1186/s12951-023-02253-y

**Published:** 2024-01-03

**Authors:** Yunyun Wang, Hao Li, Aamir Rasool, Hebin Wang, Robina Manzoor, Genlin Zhang

**Affiliations:** 1 School of Chemistry and Chemical Engineering/State Key Laboratory Incubation Base for Green, Processing of Chemical Engineering, Shihezi University, Shihezi, 832003 China; 2 Institute of Biochemistry, University of Balochistan, Quetta, 78300 Pakistan; 3 College of Chemical Engineering and Technology, Tianshui Normal University, Tianshui, 741000 China; 4 Department of Biotechnology and Bioinformatics, Water and Marine Sciences, Lasbella University of Agriculture, Uthal, 90150 Pakistan

**Keywords:** Diabetic, Polymeric nanoparticles (PNPs), Controlled release, Insulin, Oral delivery

## Abstract

Successful oral insulin administration can considerably enhance the quality of life (QOL) of diabetes patients who must frequently take insulin injections. Oral insulin administration, on the other hand, is seriously hampered by gastrointestinal enzymes, wide pH range, mucus and mucosal layers, which limit insulin oral bioavailability to ≤ 2%. Therefore, a large number of technological solutions have been proposed to increase the oral bioavailability of insulin, in which polymeric nanoparticles (PNPs) are highly promising for oral insulin delivery. The recently published research articles chosen for this review are based on applications of PNPs with strong future potential in oral insulin delivery, and do not cover all related work. In this review, we will summarize the controlled release mechanisms of oral insulin delivery, latest oral insulin delivery applications of PNPs nanocarrier, challenges and prospect. This review will serve as a guide to the future investigators who wish to engineer and study PNPs as oral insulin delivery systems.

**Graphical Abstract:**

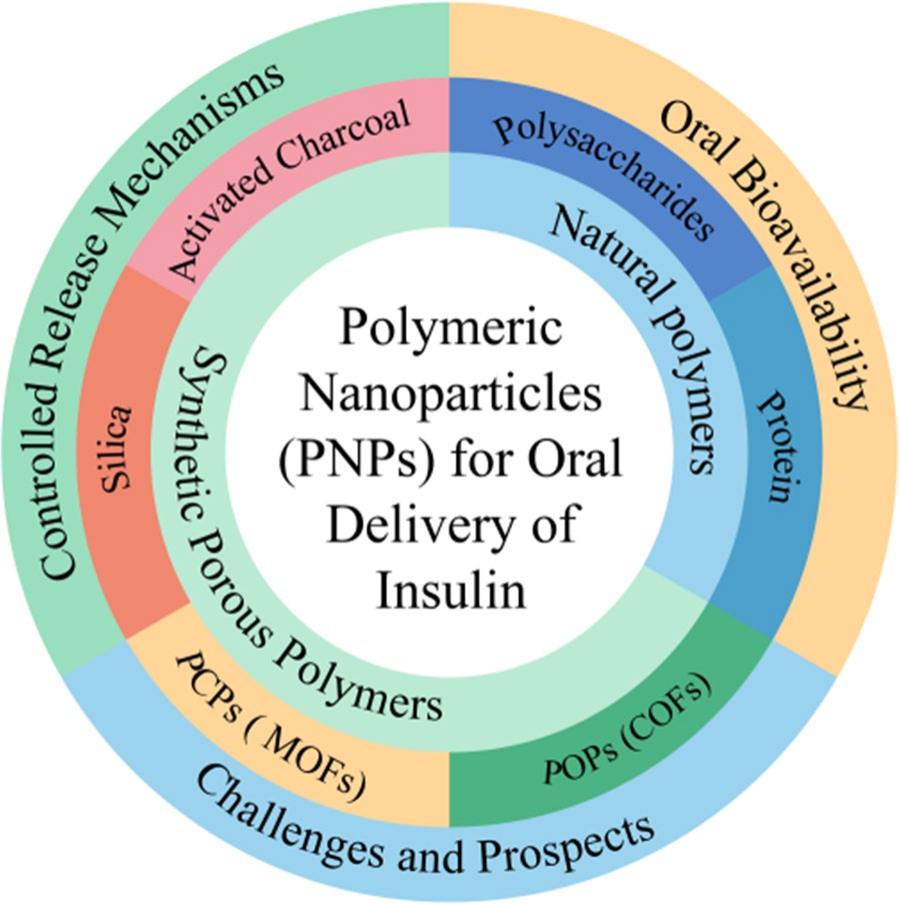

## Introduction

Insulin is a protein hormone commonly used for the treatment of diabetes, which is secreted by islet cells to regulate blood glucose. Insulin is essential for the treatment of type 2 diabetes. Diabetes mellitus is a metabolic disorder characterized by a reduction in insulin production by pancreatic islet cells, which consequently results in an elevated blood glucose level and abnormalities in the metabolism of carbohydrates, proteins, and lipids [1]. Diabetes mellitus is typically divided into two major types, type 1 diabetes mellitus (T1DM) and type 2 diabetes mellitus (T2DM). T1DM requires insulin for treatment, whereas T2DM is treated with insulin secretagogues or insulin sensitizers. Insulin treatment is only used when other medications are ineffective [2]. Notwithstanding the oral administration route of drugs with minimum invasiveness has advantages over other forms, leading to higher patient compliance [3]. Nevertheless, oral administration of insulin faces various serious challenges, such as gastrointestinal enzymes, wide pH range, mucus, and mucosal layers that restrict the oral bioavailability of insulin to < 2% [4]. At the same time, there are a number of disadvantages associated with using oral insulin, such as unwanted side effects, poor pharmacokinetics, a short distribution half-life, and so on [5].

**Fig. 1:**
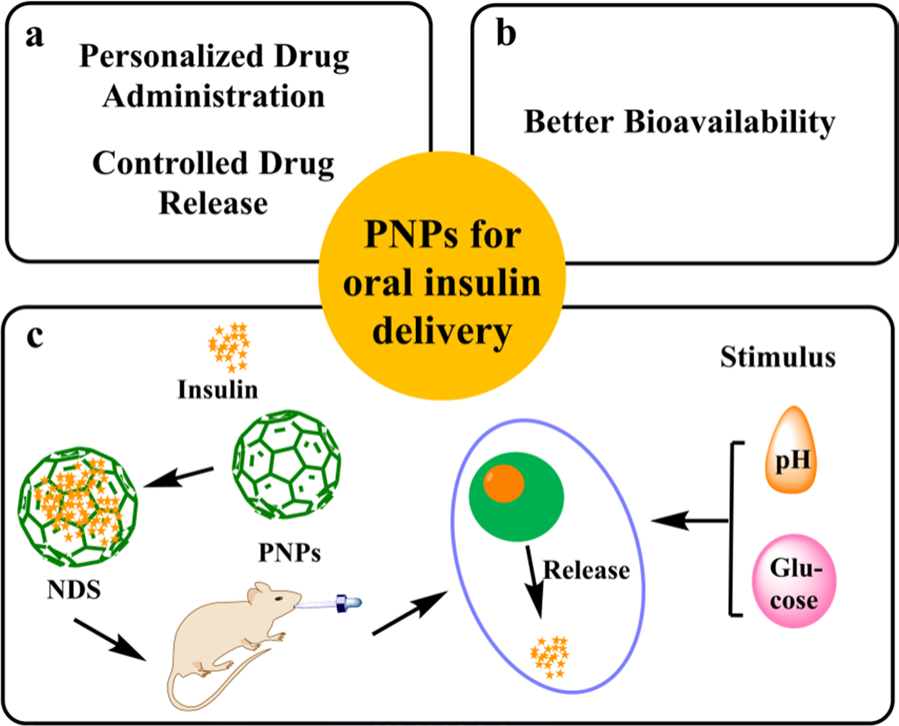
Characterization of PNPs and the mechanism of insulin release. **a**, **b** Schematic diagrams showing the unique features of PNPs. **c** Insets show details of insulin release strategies from PNPs nanocarriers (relying mainly on glucose and pH responses)

**Fig. 2:**
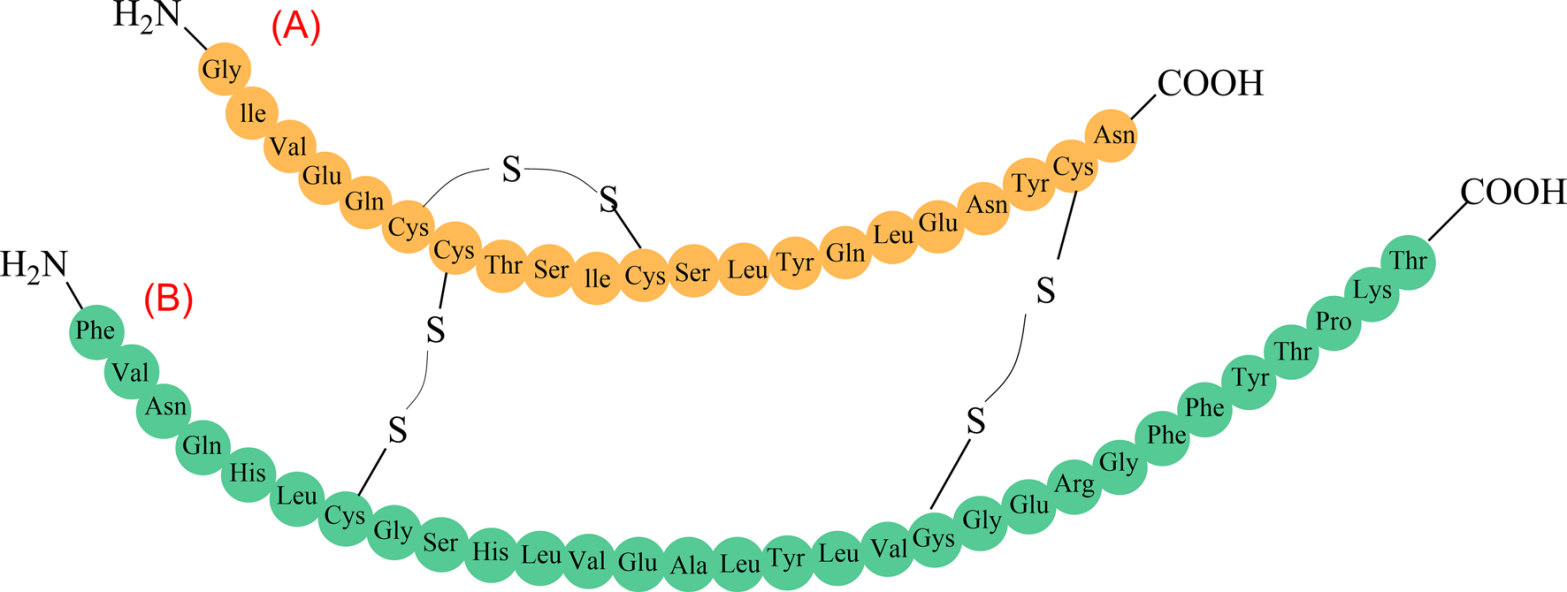
Schematic representation of human insulin. Insulin is a 51 amino acid peptide. The amino and carboxyl groups present at the ends of its polypeptide chain

The novel intelligent nanocarrier delivery systems (NDS) offer an ideal solution to address issues such as low solubility, low bioavailability, undesired side effects, poor pharmacokinetics, short distribution half-life, and many others [6]. Nanocarriers can improve insulin loading capacity, stability, selectivity, and bioavailability and reduce the toxicity and side effects of oral insulin administration [7]. Furthermore, nanopcarriers protect insulin from gastrointestinal enzymes and slowly release insulin in the digestive tract, which then enters the liver via the portal vein, where it directly participates in the glucose metabolism of liver and lowers blood glucose levels [4, 8]. Recently, many nanocarriers, including lipid-based nanocarriers (liposomes, micelles) [9–11], dendrimers [12, 13], carbon nanotubes (CNTs) [14, 15], inorganic nanocarriers (quantum dots) [16, 17], gold nanoparticles [18, 19] and polymeric nanoparticles (PNPs) [20–24] have been reported. Nevertheless, because of low loading capacity, high-cost, lack of stability, inferior biocompatibility, or bio-safety, current NPs still show some disadvantages in oral insulin delivery [9–24]. Hence, PNPs have been the most researched materials for constructing nano-based drug-delivery systems due to their unique features such as simple craft, excellent stability, preferable biocompatibility, tailored medication administration, improved bioavailability, controlled drug release from a single dosage, and drug protection until delivery to the intended place [25–27] (Fig. 1). The amino and carboxyl groups present at the ends of insulin polypeptide chain can bind with PNPs through weak bonding interactions such as π–π, CH–π, hydrogen bonding, and van der Waals interactions or covalent bonding (Fig. 2) [28–31]. These interactions remarkably enhance the stability and number of insulin molecules loaded on the PNPs.

In this review, we summarize the most recent advances in employing PNPs as promising insulin nanocarriers in the oral insulin delivery system. First, we outline the classification and applications of PNPs as stimuli-responsive polymeric nanoparticles in oral insulin delivery system. Following that, insulin release strategies from PNPs nanocarriers are explored. Finally, we present a comprehensive overview of the progress, obstacles, and opportunities of using PNPs in oral insulin delivery systems so that future researchers can engineer and investigate them in these systems.

## Drug release strategy

### Nanocarrier system

A nanocarrier is a smart delivery system that depends on the breakdown or diffusion of NPs to release the drug at the target site. All nano-based targeted delivery vehicles are made up of three basic parts: (a) a physiologically inert nanocarrier, (b) a pharmaceutically active component, and (c) a targeting moiety attached to the surface [5]. The nanocarrier systems have demonstrated in vivo their potential to boost the drug stability and selectivity while decreasing toxicity and adverse effects.

Traditional drug delivery systems (TDDS) are non-specific targeting systems that rely on the systemic delivery route to distribute drugs throughout the body. Depending on the nature of the drug, this type of system may also have numerous adverse effects and inadequate drug delivery [4]. In recent years, intelligent drug delivery systems (IDDS) have outperformed traditional delivery systems in the context of drug delivery. The drug delivery systems (DDS) regulates the therapeutic effect of drugs by addressing issues related to their solubility, bioavailability, and toxicity [32]. The DDS are intelligently designed to deliver drugs to particular organs or tissues in a regulated manner in order to keep drug concentrations within the therapeutic range [33] or even to accomplish “programmed” or “on-demand” drug release [34].

### Strategies for targeted drug release

The drug loading capacity of a nanomaterial is defined as the amount of drug docked per mass of the NPs [35], and drugs are loaded on the NP in two ways: (i) drug is combined during the NP manufacturing process, and (ii) drug is absorbed after the NPs preparation process [36]. The physical and chemical properties of a nanomaterial govern its drug loading capacity; hence, its structure must be kept intact for targeted drug delivery [37]. On the other hand, optimal size and surface charge of NPs increase their transport across the gastrointestinal epithelium. The optimized nanocarriers not only enhance the bioavailability of docked drugs but also their absorption, allowing for more effective insulin delivery [38]. The following factors largely influence drug release from NPs: pH, solubility, temperature, drug diffusion across the nanomaterial matrix, nanomaterial degradation, the drug incorporation process, and drug adsorption [39, 40]. In brief, targeted drug release relies mainly on the stimuli-responsive of the PNPs.

Takagi et al. [41] proposed the notion of stimuli-responsive material in 1990, which is about materials that respond optimally to changes in the environment while simultaneously performing their native function. According to the stimulus source, these materials may be classified as photo-responsive materials, thermal response materials, pH-responsive materials, electrically responsive materials, glucose-responsive materials, and so on. These materials have been widely employed in oil/water separation, biosensing, sustained-release drug delivery, "smart window," and so on in recent years. With each passing day, these materials are attracting the attention of an increasing number of scientists [42]. In addition, temperature-sensitive nanoparticles for insulin delivery were also of concern. Mahobia et al. [43] developed glucose and temperature-sensitive nanoparticles for insulin delivery. The released amount of insulin increased with increasing temperature from 18 to 37 ℃. However, once the drug has entered the body, it is difficult to control the temperature. The increased temperature can cause the degeneration of insulin, and somtimes, changes in temperature cannot lead to the release of oral insulin [44, 45].

### pH-responsive insulin release

In fact, ionizable polymers (polyacids or polybases) are employed to produce the nanosystems, and their cargo is released because of changes in the ambient pH, which induce conformational and/or solubility changes in polymers, resulting in the decomposition or destabilization of these nanocarriers [23, 46, 47]. Since the gastrointestinal tract’s pH varies from highly acidic (1.2–3.0) to slightly alkaline (7.5–8.0) in the stomach and intestine, respectively, it might result in pH-induced insulin oxidation and deamination, which consequently disrupt its tertiary structure and therapeutic effect. Nanoparticles can safeguard pH-vulnerable drugs from the harsh ambient of the gastrointestinal tract and thereby improve the absorption of drugs inside the gut wall, facilitate cellular uptake by particular receptors, and regulate intracellular transport pathways [4].

The insulin-carrying nanovehicles must pass through the harsh environment of the gastrointestinal tract (GIT) to allow insulin to enter the bloodstream in the oral insulin delivery systems. As a result, nanocarriers employed in oral delivery systems to carry acid-labile insulin must be stable in gastric juice (pH = 1.2) and slowly discharge insulin in intestinal fluid (pH = 6.8). In the end, it permits insulin to enter the liver via the portal circulation and lower the blood glucose levels. There are several PNPs, including polysaccharides or protein-based NPs, COFs, and others, that have demonstrated pH-sensitive insulin release for diabetes therapy [31].

Asal et al. [24] manufactured and characterized the chitosan-based nanoparticles (ChNps) before using them to deliver insulin orally to diabetic patients. In an acidic medium, ChAuNps/PLGA demonstrated significant insulin retention (96 ± 0.08%). However, a large amount of insulin was released from ChAuNps/PLGA over an extended period at higher pH levels. Mukhopadhyay et al. [48] developed pH-sensitive chitosan-alginate core shell NPs for effective and safe oral insulin administration, which can encapsulate up to ∼ 85% insulin. The insulin pH sensitivity was illustrated through in-vitro release studies in which virtually all of the encapsulated insulin was retained in a simulated stomach buffer coupled with extended release in the model intestinal environment. An in-vivo investigation demonstrated the effectiveness of CS/ALG core–shell nanoparticles as nanocarriers in an oral insulin delivery system with a substantial hypoglycemic effect and an increase of 8.11% in insulin-relative bioavailability. There was no evidence of systemic toxicity following peroral administration, indicating that these NPs could be a promising device for potential oral insulin delivery. It’s worth noting that Yang et al. [49] developed ligand-switchable NPs (Pep/Gal-PNPs) in 2022. Once orally administered, the acidic environments trigger the extension of Pep from surface in a viruslike manner, enabling Pep/Gal-PNPs to traverse intestinal barriers efficiently. Subsequently, Gal is exposed by Pep folding at physiological pH, thereby allowing the specific targeting of Pep/Gal-PNPs to the liver.

### pH and glucose dual responsive insulin release

It is critical to consider fluctuations in the pH of the GIT and the influence of blood glucose concentration on insulin release from NPs when developing oral insulin delivery systems for individuals with T1DM. Therefore, oral insulin delivery devices with dual sensitivity, like glucose and pH, would be extremely reliable. Generally, two approaches are employed to develop pH and glucose dual responsive devices: (i) either add glucose-sensitive substances (glucose oxidase (GOx), concanavalin-A, glucose binding proteins, and complex polymers of phenylboronic acid and polyol compounds) to the nanocarrier, or (ii) the nanocarrier itself has some glucose sensitivity.

Over the last few decades, many approaches for producing glucose-responsive stimuli-sensitive systems have been developed, including glucose oxidase (GOx) [50], concanavalin-A [51], glucose binding proteins [52] and complex polymers of phenylboronic acid and polyol compounds [53]. The glucose-responsive smart systems are particularly useful for patients with insulin-dependent diabetes mellitus since insulin discharge from them is controlled by blood glucose levels.

Jamwal et al. [54] developed a dextran-based glucose/pH-responsive insulin delivery system-glucose oxidase, which is clamped on acryloyl cross-aldehyde dextran NPs through the schiff base reaction, and enzymes on the NPs' surfaces hydrolyze glucose into gluconic acid, which resultantly decreases the pH of the medium and triggers the release of insulin by inducing degradation of the nanostructure. Xu et al. [55] fabricated a diabetes-specific oral delivery device by using ConA-INS-KGM NPs, which release insulin in response to different blood glucose levels. The NPs used in the development of this device were developed by cross linking konjac glucomannan (KGM) and concanavalin A (ConA).

Notably, Wei Tao et al. [56] developed an intelligent oral insulin platform based on glucose responsive polymeric nanoparticles (NPs). The NPs extended the therapeutic effect to up to 16 h in type I diabetic mice. This is the longest effective time among such types of oral insulin platforms reported to date and may reduce the frequency of insulin administration to once daily. This platform might be useful for the oral delivery of biologics in treating diabetes and related complications. In identical year, Benyettou et al. [22] manufactured imine-linked covalent organic framework (nCOF) nanostructures (TTA-DFP-nCOF) and exhibited a high bioavailability (24.1%) for oral delivery of insulin. The findings demonstrated that insulin release was minimal at pH = 2.0 and gradual at pH = 7.4. Notably, after 7.5 h of incubation, there was about 100% insulin release under hyperglycemic conditions displayed by diabetes patients (3 mg mL^−1^).

All in all, oral insulin release from NPs is mainly pH- and glucose-responsive. Table 1 shows the type of materials and their responsive conditions in different formulations.

**Table 1:** The material and their responsive condition in different formulations

PNPs	Nanoparticles	Responsive conditions	Refs.
MOFs^a^	NU-100	pH	[21]
MOFs	UiO-68-NH_2_	pH	[23]
MOFs	MIL-100	pH	[57]
MOFs	Ins-AuNPZIF-8	pH	[58]
COFs^b^	TTA-DFP-nCOF	pH and glucose	[22]
PLGA^c^	ChAuNps/PLGA	pH	[24]
PLGA/PEG^d^	Pep/Gal-PNPs	pH	[49]
Soya protein	Soya protein NPs	pH and temperature	[43]
CS^e^/ALG^f^	CS/ALG core–shell NPs	pH	[48]
GBP^g^	Hydrogel Networks	Glucose	[52]
DEGMA^h^/AAPBA^i^	p(AAPBA-b-DEGMA)	pH and glucose	[53]
Glucan	GOx^j^@NACDD	Glucose	[54]
Glucan/PLGA	Polymersomes	pH	[59]
KGM^k^/ConA^l^	ConA-INS^m^-KGM	Glucose	[55]
P(GAco-GAPBAPE)^n^/DSPE-PEG-Mal^o^	Ins/NP-Fc	pH and glucose	[56]
Starch	Hydrogels	pH	[60]
CMCD^p^/CMC^q^	CMCD-g-CMC	pH	[61]
Zein	Zein-NPs	pH	[62]
Zein/casein/dextran	A1	pH	[63]
Zein/casein/dextran	A2	pH	[63]
Zein/casein/dextran	A3	pH	[63]
Silica	SINP	pH	[64]
Silica	SINP-PEG6000	pH	[64]
Silica	SINP-PEG20000	pH	[64]

^a^Metal-organic frameworks

^b^Covalent organic frameworks

^c^Poly lactic-co-glycolic acid

^d^Polyethylene glycol

^e^Chitosan

^f^Alginate

^g^Galactose binding protein

^h^Diethylene glycol dimethacrylate

^i^Poly 3-acrylamidophenyl boronic acid-b-diethylene glycol methyl ether methacrylate

^j^Glucose oxidase

^k^Konjac glucomannan

^l^Concanavalin A

^m^Insulin

^n^Poly l-glutamic acid-co-l-glutamyl phenylboronic acid pinacol ester

^o^1,2-distearoyl-sn-glycero-3-phosphoethanolamine-n-[maleimide polyethylene glycol

^p^Carboxymethyl β-cyclodextrin

^q^Carboxymethyl chitosan

## PNPs for oral insulin delivery

The PNPs are oral drug delivery systems that are smaller than 100 nm in size, often ranging from 100 to 500 nm [65]. They are classified into two types: natural polymers and synthetic porous polymers.

### Natural polymers

Natural polymers (polysaccharides, proteins, peptides, nucleic acids, and so on) offer several benefits, including biodegradability, biocompatibility, nontoxicity, and non-immunogenicity, for encapsulating and delivering active drug components [66, 67]. Natural polymer nanoparriers are biocompatible and biodegradable; therefore, they are regarded as the most promising material for setting up the novel oral insulin delivery systems [68, 69].

It has been discovered that approximately 800 different plant species have hypoglycemic potential, and that there are a variety of additional plant active compounds that can lower blood glucose levels. As a result, the employment of natural polymers with hypoglycemia potential in the production of nanovehicles will have a dual effect on the treatment of diabetic patients [70]. Among them, polysaccharide-based drug delivery systems (e.g. glucan, chitosan, pectin, alginate, hyaluronic acid (HA), starch, cellulose, pectin, etc.) and protein-based drug delivery systems (i.e. maize, keratin, whey protein, milk protein, albumin, casein, soy protein, histone, gelatin, pulse protein, etc.) have been extensively studied [37]. Until now, the FDA has licensed two oral insulin delivery systems (ORMD-0801 and HDV-I). They have not, however, been marketed due to the volatility of their toxicity and the various adverse effects of drugs in different clinical trials [71]. Hence, more research about the characterization of natural polymer nanoparticles is required.

#### Natural polysaccharide

**Fig. 3:**
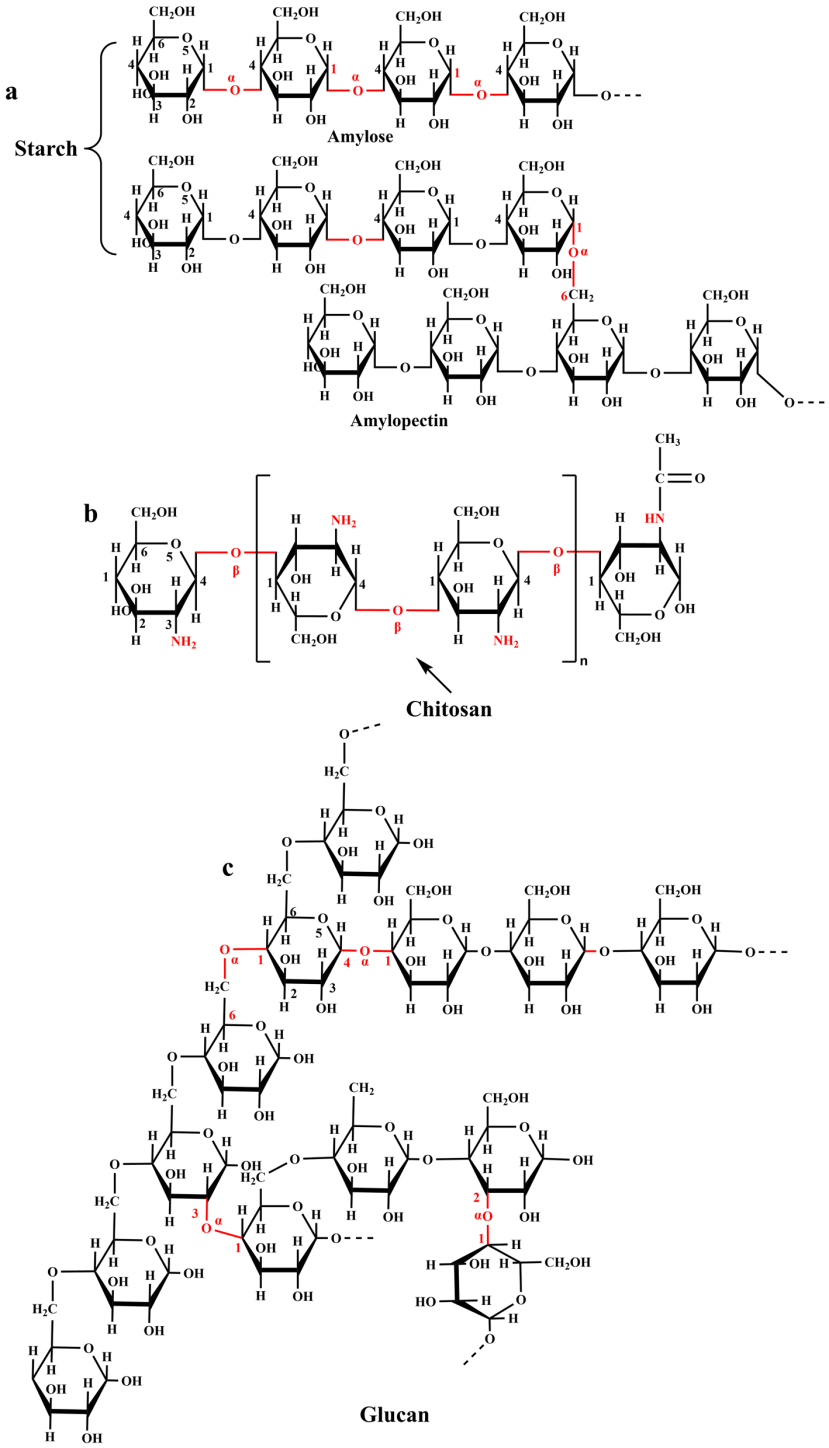
The structures of Starch (Amylose and amylopectin are found in it) (**a**), Chitosan (**b**) and Glucan (**c**)

Starch is a versatile, low-cost, and naturally occurring renewable biopolymer that contains amylose and amylopectin and may be employed in a variety of medical applications [72, 73]. Amylose is composed of α-D- (1–4) connected glucose units, whereas amylopectin is composed of the backbone of α-D- (1–4) joined glucose units and certain α-D- (1–6) linked branches [74] (Fig. 3a). Natural starch, on the other hand, has drawbacks like high digestibility and swelling index, high glycemic index, quick enzymatic breakdown in biological systems, and many others [75]. However, modified starch can alleviate some of the drawbacks associated with native starch. The modified starch derivatives (carboxymethyl short-chain amylose, cyclodextrin (CD), and others) have several potential therapeutic applications due to their low toxicity, high biocompatibility, and exceptional biodegradability [60]. Meneguin et al. [66] studied the employment of modified starch derivatives in oral insulin delivery systems. Yang et al. [61] on the other hand, produced carboxymethyl chitosan hydrogel-based carboxymethyl cyclodextrin-grafted microparticles for oral insulin delivery. The study's findings indicated that insulin was successfully retained by the hydrogel in the gastric environment and slowly released in the GIT. The smart polymeric hydrogel was created by EDC coupling NHS technique from precursor carboxymethyl chitosan (CMC) and carboxymethyl cyclodextrin (CMCD).

Chitosan (CS) is a non-toxic, cationic, linear, heteropolysaccharide derived from deacetylated chitin and composed of d-glucosamine and n-acetyl-d-glucosamine monomers linked by β-(1–4) glycosidic bonds [39, 76, 77]. The amino functional groups in CS play a vital role in regulating biochemistry and electrostatic interactions in nanodrug delivery systems, as well as offering solubility in slightly acidic solutions. [77, 78] (Fig. 3b). Chitosan's sticky properties and electrostatic attraction to negatively charged mucus are due to positively charged acetylglucosamine units [79]. CS is also biodegradable and cheap, making it an ideal polymer for oral drug delivery [80]. The current research has indicated that CS NPs are the best nanovehicles for oral administration of insulin to diabetic patients due to their perfect properties, such as biocompatibility, biodegradability, bioavailability, encapsulation rate, noncarcinogenicity, and nontoxicity.

Dextran is a branched chain homopolysachride composed of glucose monomers linked by α-1,6 glycosiside bonds and branched at positions α-1,2, α-1,3, and α-1,4 (Fig. 3c). The gluan-producing strains regulate the length and nature of branching units at positions 2, 3, and 4. Dextran is primarily synthesized from sucrose by numerous *Lactobacillus* species via glucan sucrase, which catalyzes the conversion of d-pyran glucose residues from sucrose to glucan [68]. Dextran, unlike other polysaccharides, is poorly degraded by regular amylases such as salivary amylase or malt amylase. Only dextranase in the lumen of the large intestine, liver, spleen, and kidney can depolymerize dextran [68, 81]. With these facts in mind, the dextran-based oral insulin delivery system will safeguard insulin against chemical and enzymatic degradation in the gastro-intestinal tract, as well as promote absorption by the intestinal epithelium, resulting in improved oral bioavailability [68]. The dextran ester, dextran ether, and dialdehyde dextran are the most prevalent dextran derivatives, with the latter being employed for oral insulin administration due to its exceptional properties.

Alibolandi et al. [59] developed an oral insulin delivery system based on the glucan-poly (propylene ester-hydroxyl-acetate copolymer) PLGA copolymer, which displayed sustained insulin release in a GIT simulation condition. Jamwal et al. recently developed a novel glucan-based glucose- and pH-responsive insulin delivery system [54], and its insulin release property as well as release kinetics under different pH mediums (AGF, pH 1.2, and AIF, pH 7.4) were studied using mathematical kinetic models. The insulin-loaded NPs were then tested in vitro for insulin release in response to various glucose concentrations.

#### Natural proteins

Proteins are one of the most significant natural biomaterials that are used to construct the novel nanovehicles because they display unique characteristics such as biodiversity, biodegradability, low immunogenicity, non-toxicity, and biocompatibility [82, 83]. Since protein-based nanovehicles possess a well-defined primary structure and can also be tailored by pre- or post-functionalization due to this property, different drugs, various components, and carriers can be integrated with hydrophobic or hydrophilic domains of proteins by using different reagents. Protein nanoparticles will also provide energy to the diabetic patient in addition to acting as insulin delivery vehicles [43]. Soy protein, albumin, transferrin, zein, keratin, and other proteins are exploited in the synthesis of nanopores, although transferrin, zein, and keratin have gained significant attention in the last decade as prospective multifunctional biopolymers [84].

Zein is a natural GRAS (Generally Recognized As Safe by the FDA) protein produced in maize that is not only extensively applied in the pharmaceutical industry but also in the production of polymer nanocarriers due to its safety, biodegradability, and nontoxicity. Furthermore, zein NPs have the potential to significantly enhance the oral bioavailability of small and large molecules. Inchaurraga et al. [62] developed naked zein NPs and polyethylene glycol-coated nanoparticles as oral insulin vehicles, which displayed bioavailability of 4.2% and 10.2%, respectively. The results of Inchaurraga et al. displayed that zein possess certain amount of insulin-carrying capacity. Bao et al. [63] developed insulin- and cholic acid-loaded zein NPs with dextran surfaces to increase insulin oral absorption in the GIT and the liver, the principal action organ of endogenous insulin. In type I diabetic mice, NPs showed an insulin loading efficiency of 74.6% and an oral pharmacological bioavailability of 12.5–20.5% [63].

Keratin is a structural protein present in mammals (hair, nails, skin, wool, hooves, and horns) and birds (bills and feathers) [83, 84]. Kunjiappan et al. [85] recently focused their research on the utilization of human hair keratin as carriers of indoloquinoxaline (INDX) derivatives for the treatment of type 2 diabetes mellitus. Indoloquinoxaline derivatives exhibited a variety of biological functions, including anti-diabetic potential, and the incorporation of keratin significantly increased their insulin encapsulation efficiency and substantially increased the release capacity. It has been demonstrated that keratin has the ability to load insulin. A large number of studies have shown that activation of adenosine monophosphate-activated protein kinase (AMPK) and suppression of the activity of protein tyrosine phosphatase 1B (PTP1B) are effective treatments for T2DM. Therefore, Ferroni et al. [83] formulated and manufactured INDX derivatives and incorporated keratin NPs using the dispersion method. The encapsulation efficiency and loading capacity of INDX into the keratin carrier were 89.9% and 11.9%, respectively. The results show that keratin is a promising choice for acting as a carrier for the oral insulin delivery system, but additional study is needed to validate it.

Transferrin (Tf) is a protein produced by the human body that regulates iron absorption and transportation by binding to TfR on epithelial cells. It is also resistant to chymotrypsin and tryptic digestion [23, 86]. Hence, when a large molecule of transferrin binds to a smaller molecule of insulin, it protects the insulin from digestive enzymes by preventing access to the insulin [87, 88]. Therefore, the use of Tf-modified NPs to guard insulin against GIT breakdown is crucial for oral insulin administration. Tf-coated polymeric NPs for oral insulin administration were developed by Zhu et al. and the surface-loaded Tf improved their transepithelial transport via receptor-mediated transcytosis.[89]. Nevertheless, these NPs showed poor release of insulin prior to transepithelial transport and limited their therapeutic bioavailability to ~ 7.8%. It is surprising that Wei Tao et al. [90] developed an oral hydrogel microbeads-mediated in situ synthesis of selenoproteins. We can start with the self-designed protein in vivo of PNPs that can survive the harsh environment of the gastrointestinal tract for oral insulin delivery. It can not only solve the disadvantage of natural protein that is easily degraded by gastrointestinal enzymes, but also solve the bio-safety of PNPs in vivo.

### Synthetic porous polymers

The benefits of synthetic porous polymers (SPP) over natural polymers and other material types include the flexibility of producing polymeric materials and the potential of synthesizing this material according to pathology and patient demands [26]. The examples of SPP include charcoal activation, silica, porous coordination polymers (PCPs, including MOFs), porous organic polymers (POPs), and many others [91]. POPs are mostly composed of hyper-cross-linked polymers (HCPs), polymers with intrinsic microporosity (PIMs), conjugated microporous polymers (CMPs), porous aromatic frameworks (PAFs), covalent organic frameworks (COFs), and many more. POPs possess a distinct pore structure, variable pore size, tunable structure, and surface-modifiable characteristics [92].

The synthetic polymers MOFs [21] and COFs [22] have been shown to increase the bioavailability and delayed release of insulin in oral insulin administration systems [68, 93].

#### Activated charcoal

Activated charcoal has been shown in several studies that it prevents the obesity, metabolic syndrome, and a variety of other illnesses. Zhang et al. [93] discovered that combining acidic activated charcoal with an HFD reduced obesity in insulin resistance mice in a dose-dependent fashion However, because activated charcoal is often alkaline and can be influenced by gastric juice, it may not be able to protect insulin against pepsin breakdown. On the other hand, because activated charcoal is found in the form of powder, it is not digested and hence might affect the digestive tract [94].

#### Silica

Insulin can be administered orally using promising silica nanoparticles (SNs), particularly mesoporous silica nanoparticles (MSNs) due to their high biocompatibility, exceptional encapsulation efficiency, and controlled release, as well as appropriate loading processes [95]. Andreani et al. [64] synthesised SiNP and PEG-SiNP using the sol–gel technique and investigated them as insulin carriers. Later, Juère et al. [96] created dendritic mesopores of DMSNs in which insulin was effectively encapsulated, resulting in the prevention of insulin release in the stomach (pH = 1.2) and increased insulin transport to the intestine (pH = 7.4).

However, SNs have two major drawbacks: (i) poor epithelial membrane penetration, which has been greatly improved by necessary functionalization, and (ii) the complicated synthesis process involved in the production of SNs, which restricts their scale-up production. [95]. As a result, further research is required before using the silica nanoparticles in oral insulin administration.

#### PCPs

PCPs (including MOFs) are materials having an infinite network structure formed by the coordination bonding of metal nodes (metal ions or metal clusters) and organic linkers [97]. Contrary to traditional inorganic nanostructures, which are generally not biodegradable, many PCPs have intrinsic biodegradability and may be excreted by the kidneys without long-term retention in the body, reducing concerns about their possible long-term toxicity. It is evident that PCPs are a promising class of nanomaterials used in the field of nanomedicine [98], and some progress has already been made in employing MOFs in oral insulin delivery systems. It has been widely employed in major biomedical applications, including medication administration, biomedical imaging, and photodynamic therapy (PDT). It is important to mention here that ˃ 20,000 MOFs with various topologies have been registered [99–101]. These MOFs were produced using various synthetic techniques. Among these MOFs are IRMOFs (isoreticular metalorganic frameworks), ZIFs (zeolitic imidazolate frameworks), CPLs (coordination layered layers), MILs (materials of the Institute Lavoisier), UiOs (University of Oslo), and others [99–102].

### Synthesis strategy and functionalization of MOFs

1. Synthesis strategy of MOFs

MOFs are generally manufactured in a single step through the use of suitable metal salts, organic ligands, and solvents [103]. MOF synthesis was performed out at medium and low temperatures. In addition to solvothermal, reverse micro-emulsion, and template approaches, microwave, direct precipitation, and sonochemical (ultrasonic) technologies have recently been developed [104].

2. Functionalization of MOFs

**Fig. 4:**
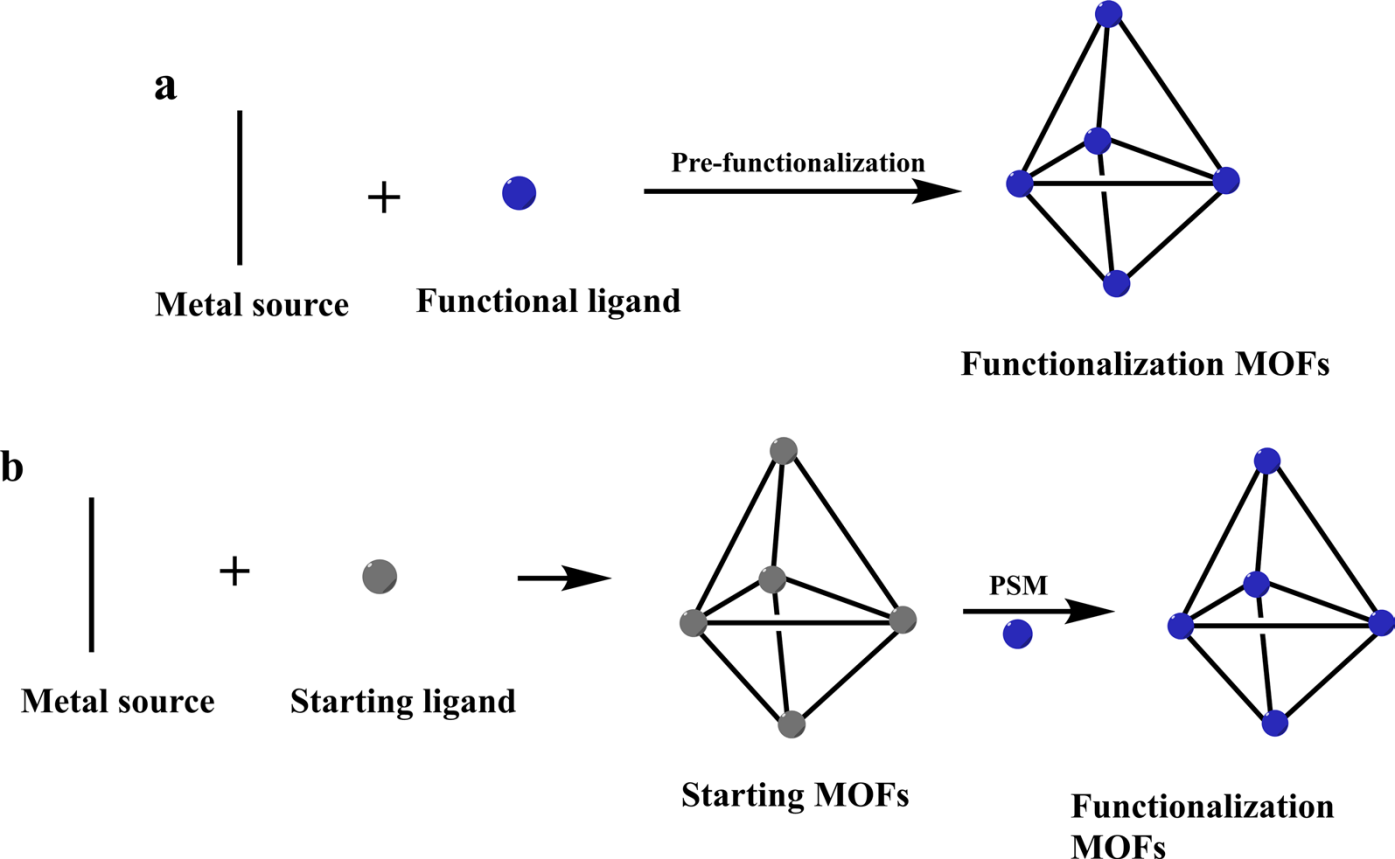
Schematic diagrams of prefunctionalization and PSM of MOFs. **a** Pre-functionalization: Functional ligands are made by substituting the organic ligand with specified substituents and then directly incorporating the changed ligand into MOFs for functionalization. **b** PSM: Starting MOFs synthesized using unmodified organic ligands, which were then functionalized by adding new functional groups

MOF functionalization can be divided into two types: pre-functionalization and post-synthetic modification (PSM) [105]. Pre-functionalization is the process of incorporating functional groups into the structure of MOFs prior to synthesis, which is accomplished by changing the organic ligand with specified substituents and then directly employing the changed ligand in the MOFs necessary for solvothermal synthesis (Fig. 4a). PSM is the method of adding new functional groups to a material after it has been synthesized, rather than on its molecular precursors (Fig. 4b). Hence, the compatibility between newly introduced functional group, reaction conditions, and final material produced through PSM is necessary. The incompatibility with the synthesis method must be avoided to generate the materials with distinct functionalization and structural stability. PSM offers an advantage over the pre-functionalization method due to better control over the type and number of functional groups added. As a result, when it is difficult to obtain MOFs of interest via the pre-functionalization method, this route is often taken for chemical modification. There are many kinds of post-synthetic modifications, including: covalent functionalization, coordinated covalent modification, surface functionalization, post-synthetic metalation, post-synthetic exchange, modification after coordination modification, dis-protection after synthesis, polymerization after synthesis, and cluster modification after synthesis.

### Research progress in oral insulin delivery of MOFs

MOFs have many unique properties for drug delivery systems (DDS) when compared to traditional materials. MOFs, for example, not only have large porous surface areas that aid in drug or guest molecule loading efficiency, but they are also very easily functionalized due to their excellent biocompatibility, water solubility, and biodegradability, all of which improve drug bioavailability and efficiency in the body. Additionally, drugs may be chemically coupled or physically encapsulated within the carriers via a variety of interactions such as hydrogen bonds, Van der Waals forces, the π–π effect between aromatic rings, electrostatic interactions, coordination bonds, covalent bonds, and etc. The biomolecules are usually coupled to the MOFs via using different methods, such as surface attachment, covalent linkage, pore encapsulation, in situ encapsulation, forming bio-MOF (metal–biomolecule frameworks). The outstanding encapsulation capability of MOF makes it a one-of-a-kind platform for drug loading, therefore, several drugs, including DOX, 5-Fu (5-fluorouracil), β-estradiol, and insulin, have been delivered using this technique [106]. In recent years, the researchers have synthesized a variety of MOFs for of oral insulin delivery. Furthermore, several researchers conducted in vivo studies in mice and showed that MOFs have higher bioavailability in oral insulin administration.

Chen et al. [21] devised an acid-resistant Zr-based MOF (NU-1000) insulin delivery device with pores that interact well with insulin and disintegrate in the presence of phosphate ions (as in blood). In approximately 30 min, the NU-1000 was able to load 40% wt insulin via post-synthetic pore encapsulation process. In a simulated gastrointestinal environment, insulin-loaded NU-1000 was stable and rapidly released insulin under physiological settings (pH 7.0 in PBS). Zhou et al. created a polymer microsphere system that encapsulated insulin and SDS in MIL-100 [57]. The polymer coating of methoxy poly(ethylene glycol)-block poly(l-lactide) protected the MOF from degradation in the gastrointestinal environment, while SDS increased permeability through the intestinal membrane. The MIL-100 NP enhanced the endocytosis of insulin in monolayer Caco-2 cell culture and displayed excellent monolayer permeability. This oral delivery method, at 50 IU/kg, attained a maximal plasma insulin level (50 mIU/mL) at 4 h, and it remained higher for 8 h in a BALB/c mouse type I diabetes model. This system reduced glucose levels more slowly and for longer than subcutaneous injections. Moreover, the accumulation of insulin in the liver revealed that insulin discharged from the MOF moved through the portal veins to the liver and then to the cardiac tissue, closely resembling endogenous insulin circulation patterns. Rohra et al. demonstrated that the microfluidic synthesis of a metal–organic framework (MOF) (Ins-AuNPZIF-8) for insulin administration is based on the glucose stimulus response [58]. The insulin loading per unit weight of MOF was determined by using size exclusion chromatography and high-performance liquid chromatography, and the results were 77 and 88%, respectively, in the batch and microfluidic methods. The drug release experiments validated the MOFs’ response to glucose, which caused insulin release. This is the first work in which a SAR micromixer is used to synthesise MOFs, particularly ZIF-8, to encapsulate insulin and AuNPs. Simultaneously, Zou et al. encapsulated an adequate amount of insulin in acid-resistant metal–organic framework nanoparticles (UiO-68-NH2) and coated the exterior with targeting proteins (transferrin) to achieve very effective oral insulin administration [23]. The transferrin-coated nanoparticles efficiently crossed the intestinal epithelium and released the insulin under physiological settings, which resulted in a significant hypoglycemic response and 29.6% oral bioavailability.

The researchers have demonstrated that MOF nanoparticles can protect proteins from the degradative environment of the stomach and intestine, allowing them to be used in oral macromolecule delivery systems.

#### POPs

POPs offer higher surface areas and more controlled size distributions than traditional porous materials (such as charcoal activation, silica, and PCPs), making them appropriate for oral insulin administration systems.

The drug and the carrier are linked through weak interactions like van der Waals forces, hydrogen bonds, and hydrogen bond interactions [107–109]. POPs are mostly composed of hyper-cross-linked polymers (HCPs), polymers with intrinsic micro-porosity (PIMs), conjugated microporous polymers (CMPs), porous aromatic frameworks (PAFs), covalent organic frameworks (COFs), and other substances [91]. The COFs are very crystalline, whereas HCPs, PIMs, CMPs, and PAFs are amorphous [109]. The COFs, being a distinct class of porous materials with distinct properties, are regarded as having potential use in oral insulin delivery among POPs. The COFs are crystalline organic porous polymers made up of light elements such as C, H, O, N, and B that form covalent bond interactions via reversible reactions. COFs are low-density and high-surface-area nanomaterials that also have the following characteristics: customizable pore size, well-ordered internal structures, different topologies, and diverse monomer sources, which provide them with outstanding modifiability and a wide range of functions [110]. COFs materials have been extensively employed in adsorption and separation of biological macromolecules and gas [111–118], heterogeneous catalysis [119, 120], fluorescent sensing [121–125], proton / ion conduction [126, 127], accumulation energy [128, 129] and drug loading and release [130–137]. Côté et al. [138] designed and synthesized the first COFs from phenyl-di-borate [C_6_H_4_[B(OH)_2_]_2_ and hexa-hydroxyl-benzyl-benzene [C_18_H_6_(OH)_6_] with condensation reactions. Over the last decade, researchers have developed and synthesized a wide range of COFs and applied them to a variety of areas. COFs have gradually developed into an important class of POPs. On the basis of their topology, they are classified into two-dimensional (2D) and three-dimensional (3D) COFs, and based on different linking methods, they are divided into Boroxine, Boronate, Imine, Imide, Hydrazone, Azine, Triazine, Beta-ketoenamine, and so on.

### Topological design of the COFs

Topological graphs are employed to design the COFs, which compress the knots and connectors of different geometries to form extended polygonal structures. However, different topological graphs are required for the designing of 2D and 3D COFs. The monomers can be classified into C1, C2, C3, C4, C6, and Td geometry based on their geometry, with Td geometry being required for the creation of 3D COFs [139].

**Fig. 5:**
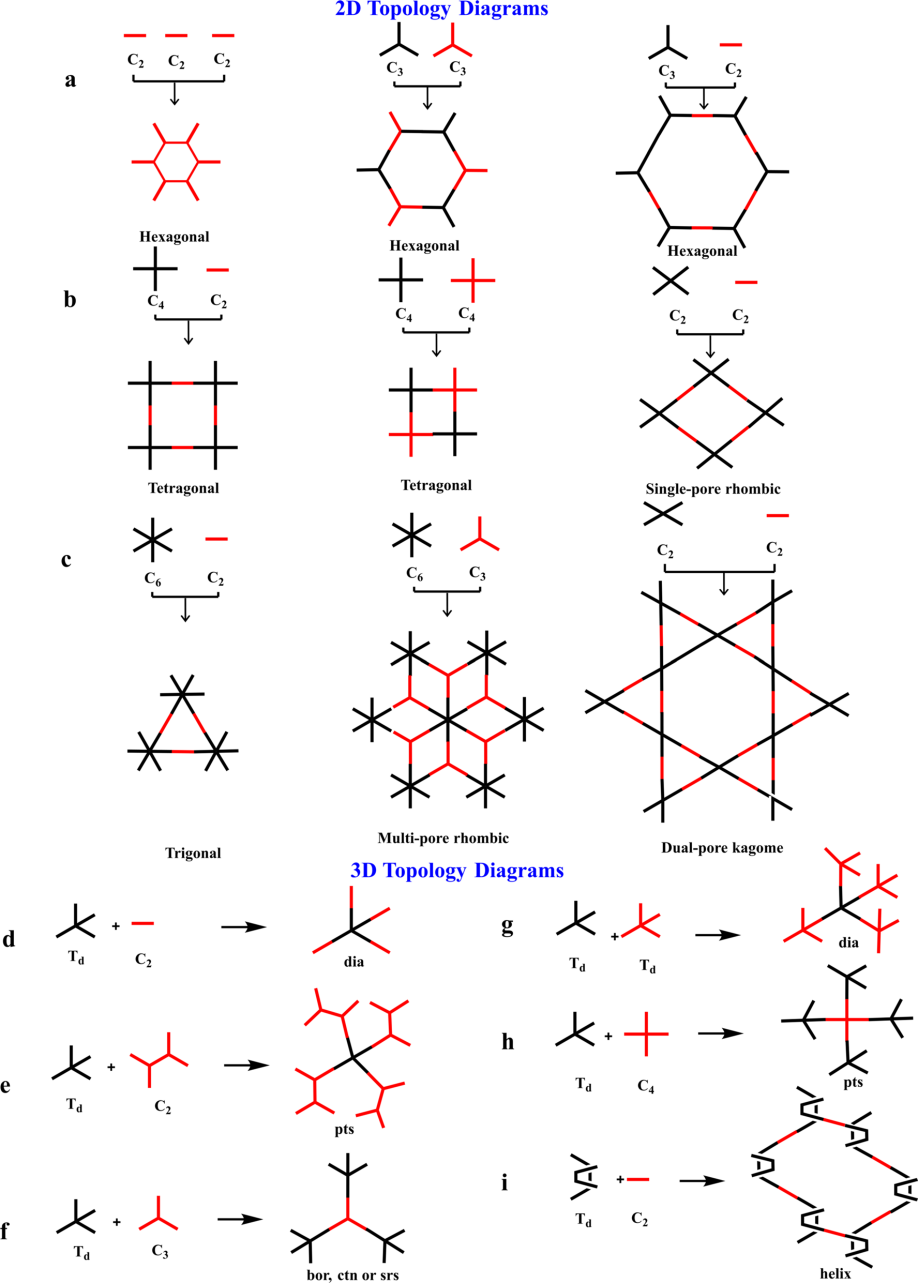
Topology diagrams for designing 2D and 3D COFs to create skeletons and pores different. **a**) Hexagonal COFs formed either by self-condensation of C2-symmetric units or combination of C3-symmetric vertices and C2-/C3-symmetric units. **b**) Tetragonal COFs formed based on the C4+C2 topology or C4+C4 topology. Four-armed C2 vertices and linear C2 linkers lead to the single-pore rhombic shape. **c**) Trigonal COFs built by C6-symmetric knots and C2-symmetric linkers; multi-pore rhombic COFs constructed by C3-symmetric vertices and C6-symmetric units; dual-pore kagome topology constructed by four-armed C2 vertices and linear C2 linkers. The dia network formed by **d**) [Td + C2] or **g**) [Td + Td] diagrams. The pts network formed by **e**) [Td + C2] or **h**) [Td + C4] diagrams. The bor, ctn or srs network formed by **f**) [Td + C3] and the helix network formed by i) [Td + C2] diagrams

**Fig. 6:**
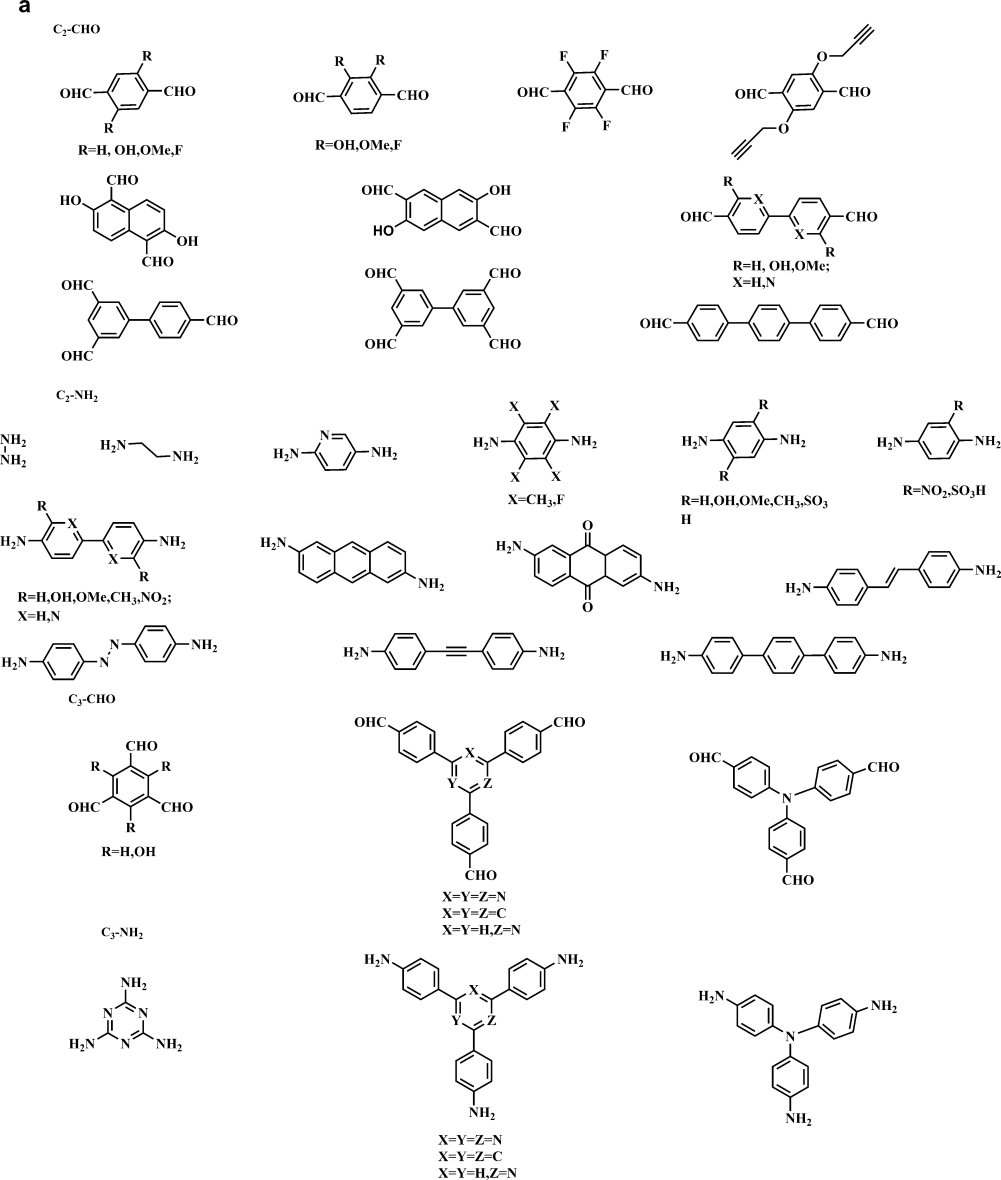

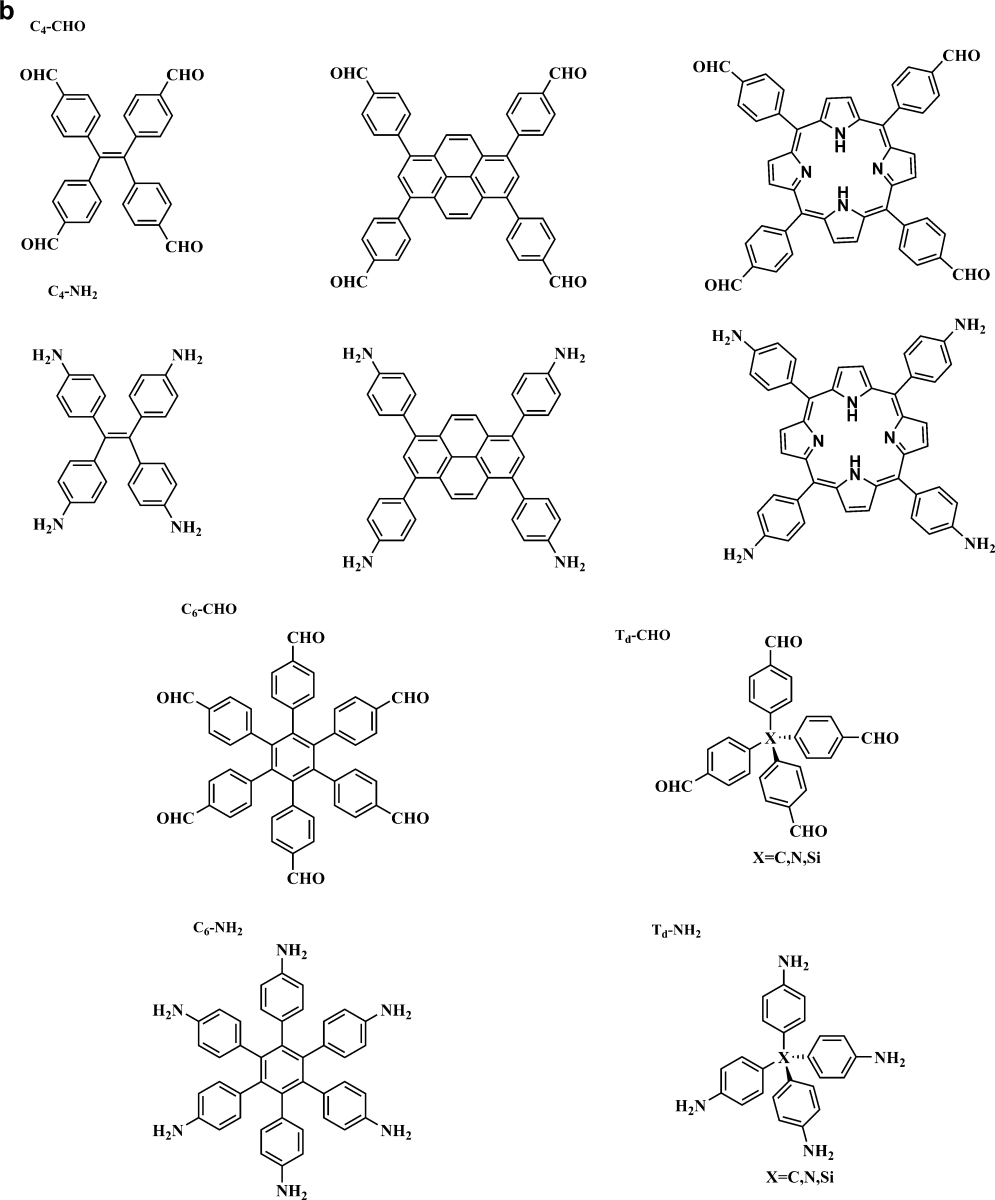
Typical examples of C2, C3, C4, C6, and Td symmetric monomers with aldehyde functional groups via Schiff-base chemistry for the synthesis of COFs. **a**) C2-, C3-NH_2_ and -CHO building blocks. **b**) C4, C6, Td-NH_2_ and -CHO building blocks

Monomers having C1, C2, C3, C4, and C6 geometries are used to produce 2D COFs, whereas C2, C3, C4, and C6-symmetric monomers act as knots. The combination of [C2 + C2 + C2] (Fig. 5a) permits the construction of hexagonal 2D COFs in the self-condensation event, and this is mostly employed for the construction of boroxine and triazine-linked frameworks. The [C3 + C2] and [C3 + C3] (Fig. 5a) combinations yield hexagonal 2D COFs in two-component [1 + 1] poly-condensation systems with one knot and one linker, whereas [C4 + C2] and [C4 + C4] (Fig. 5b) pairings produce tetragonal 2D COFs. The [C2 + C2] (Fig. 5b) and [C6 + C2] (Fig. 5c) combinations yield rhombic and trigonal 2D COFs, while [C6 + C3] (Fig. 5c) produces multi-pore rhombic 2D COFs and [C2 + C2] (Fig. 5c) also form the dual-pore kagome-type 2D COFs. The knot unit with Td or orthogonal symmetry is essential to grow the backbones of 3D COFs in a 3D manner. However, various degrees of interpenetration are present in 3D COFs, including dia {[Td + C2] (Fig. 5d) and [Td + Td] (Fig. 5g)}, pts {[Td + C2] (Fig. 5e) and [Td + C4] (Fig. 5h)}, ctn {[Td + C3] (Fig. 5f)}, bor {[Td + C3] (Fig. 5f)}, srs {[Td + C3] (Fig. 5f)} and helical structures {[Td + C2] (Fig. 5i)}. The Figs. 5 and 6 describe the essential topological diagrams and building blocks for the construction of 2D and 3D COFs using Schiff-base chemistry.

The topology diagram provides the principles for designing 2D and 3D COFs and provides a foundation for the COF field. A COF's structural diversity depends on its topology diagrams and building blocks.

Since the hexagonal topology diagram was developed, other shapes have been added, including tetragonal, rhombic, trigonal, and kagome. A large number of building blocks have been developed, ranging from simple arene to large π electron donors and acceptors, coordination sites, catalytic units, and spin systems. Linkages have also changed from non-conjugated to partially and fully conjugated. Moreover, the lattice was converted from isotropic to anisotropic. It is through these changes that the structural diversity of COFs is greatly enhanced, and the range of their properties, functions, and applications are greatly expanded [139].

### Synthesis and functional regulation of COFs

1. Synthesis of COFs

Solventothermal synthesis, microwave synthesis, interface synthesis, mechanical grinding synthesis, the sonochemical method, heating reflux synthesis, room-temperature synthesis (currently only for COFs with imine-linked -linked atoms), ionothermal synthesis, and other methods are used to create COFs. The solvent thermal approach is one of the most frequent and gentle procedures, although the synthesis period is lengthy, taking 2–7 days. As a result, developing moderate and short synthesis procedures is critical.

de la Peña Ruigómez et al. [140] developed a simple, quick, and efficient Schiff-base reaction through which RT-COF-1 was synthesized at room temperature from 1,3,5-Benzenetricarboxaldehyde (TFB) and 1,3,5-Tris(4-aminophenyl)benzene (TAPB) in the presence of acetic acid and inter-phenol, dimethyl sulfoxide (DMSO). Guan et al. [141] developed a technique for the synthesis of COFs that employed ionic liquids to produce 3D-IL-COF-1, 3D-IL-COF-2, and 3D-IL-COF-3. The 3D-IL-COF-1 was manufactured in ~ 3 min at ambient temperature and pressure, proving that COFs can be produced chemically.

2. Functional regulation of COFs

The functions and features of COFs are generally lies in their pore structure and functional groups. The functionalization of materials may occur in three ways: bottom-up incorporation of functional moieties, in situ functionalization, and post-modification [142].

- Bottom-up incorporated functional moieties

This approach involves modifying the functional groups on the structural components prior to COF synthesis, because of which all functional groups are uniformly distributed in the COF framework and the number of active sites can be precisely regulated. When the modified functional groups on the structural units have a high steric hindrance then crystallization of the COFs decreases, but even it becomes difficult to perform.

b. In situ functionalization

The in situ functionalization method is performed to incorporate the particular functional groups at specified locations on the structures of COFs during the condensation process. This method is relatively simple, but the applications of the COFs produced by it are limited.

c. Postmodification

The post-modification process involves synthesising the initial framework of functional groups with reactivity as structural units and then grafting other types of functional groups into the pore tract. Although the synthesis of COFs by this method is simpler than that of bottom-up-incorporated functional moieties, the structure and porosity of these materials may be damaged during the modification process, and in addition, the quantity and distribution of active sites are difficult to manage.

As a result, it is critical that we develop moderate modification methods over the next decade.

### Research progress in oral insulin delivery of COFs

COFs display a high drug loading capacity and high drug delivery efficiency, because of their unique characteristics, such as a high specific surface area, excellent crystallinity, a porous structure, and outstanding biocompatibility; consequently, they have extensive application possibilities in the field of drug delivery. The imine, imide, hydrazone, and triazine-type COFs are most commonly employed for drug delivery, but other types of COFs have also been used, such as phenazine linkage and olefin linkage. These COFs have a higher nitrogen content whose lone pair electrons are used to load drugs via hydrogen bonds or π−π bonds, and they significantly increase the drug-loading rate of COFs [143]. Fang et al. [130] developed two porous polyimide COFs (PI-COFs) in 2015 and used their large, uniform porosity and high stability to accomplish loading and controlled release of the model drug ibuprofen (IBU). This work is the first to demonstrate the use of COF materials in drug delivery. Their pioneering work has paved the way for the application of COFs to the field of drug delivery, and a number of additional functionalized COFs were developed to boost drug delivery efficiency in nanodrug delivery systems (NDDSs) [130–137, 144].

In 2016, two different nanoscale porous COFs were employed to load an anticancer drug (5-fluorouracil (5-FU)) by Bai et al. [145]. These drug loaded COFs showed good dispersibility in aqueous solution, high drug loading efficacy (the drug loading efficacy reached up to 30 wt%) and complete release in vitro. The same year, Vyas et al. [146] synthesised triazine COFs (TTI-COF) and used them for the quercetin uptake and release, highlighting the potential of COFs as nanocarriers. In 2017, Mitra et al. [147] modified TpASH by post-synthesis modification to yield folate conjugated CONs (TpASH-FA) for targeted drug delivery of anticancer drug 5-fluorouracil (5-FU) within the breast cancer cells MDA-MB-231. In 2018, Zhang et al. [148] developed a facile synthesis of a polymer-COF nanocomposite (denoted as PEG-CCM@APTES-COF-1) and targeted drug delivery of DOX. The results showed an enhanced tumor-inhibition effect was observed compared to the free DOX formulation. Wang et al. [149] prepared a style of pH-responsive COPs (THPP-BAE-PEG COPs), which can encapsulate anticancer drug doxorubicin (DOX) with the pH triggered drug release for chemotherapy. In 2020, Liu et al. [150] developed PEGylated redox-responsive nanoscale COFs (denoted F68@SS-COFs) for efficiently loading and delivering doxorubicin (DOX), showing a very high DOX-loading content (about 21%). In 2021, Anbazhagan et al. [151] designed a thioether-terminated triazole bridgecontaining COF (TCOF) for pH and glutathione S-transferase (GSH) dual-sensitive anticancer drug (DOX) delivery. Notably, almost 70%-80% of the loaded drug was released in 72 h of incubation.

Despite the fact that COFs have always been studied for drug delivery, it is probable that none of them have been used in oral insulin trials. Benyettou, et al. [22] presented an imine covalent organic framework (TTA-DFP-nCOF) that was first time used for the construction of oral insulin delivery systems in 2021. The triazine-based TTA-DFP-nCOF was chosen for its strong stability under harsh conditions, including acidic environments. This delivery system is distinguished by its excellent insulin-loading capacity (65 wt%), biocompatibility, bioavailability (24.1%), and insulin protection under severe circumstances, and hyperglycemia-induced drug release. The estimated pore size is roughly 1.7 nm, which is substantially smaller than insulin's molecular size (2.5–3 nm). As a result, it was concluded that insulin was intercalated between the layers of COF rather than deposited in the pores. The insulin orally delivered to the body is immediately absorbed by the intestinal epithelium and enters the liver via the portal vein, allowing glucose homeostasis to be maintained. Diabetes is known to induce damage to the liver and kidney, but no alterations were observed in these organs in TTA-DFP-nCOF/insulin-treated rats, demonstrating that the particles are harmless. Furthermore, it improved liver and kidney function, which suggested that COFs offer significant promise for oral insulin administration.

In conclusion, PNPs play a significant function as nanodrug carriers and show promising outcomes in the treatment of diabetes. Table 2 summarizes more important studies.

**Table 2:** PNPs nanoparticles for the entrapment of insulin and used against diabetes

PNPs	Nanoparticles	Particle size (nm)	EE(%)^a^	LC(%)^b^	Bioavailability (%)	Zeta potential (mV)	Refs.
Natural polymers nanocarriers							
Starch	IN^c^-Z-CSA/CS	200.77 ± 6.47	90.5 ± 1.2	9.2 ± 0.2	15.19	− 40.06 ± 1.04	[152]
starch/ALG^d^	In-Alg	/	64.14	/	/	/	[153]
starch/ALG	In-MS-Alg	/	92.34	/	/	/	[153]
starch/ALG	In-WS-Alg	/	93.79	/	/	/	[153]
starch/ALG	In-RS-Alg	/	80.14	/	/	/	[153]
β-CD	NAC-HP-β-CD-Arg@insulin	/	/	/	9.06	/	[154]
β-CD	CMCD-g-CMCs	/	/	30–31.9	/	/	[60]
CS	CS	216.2 ± 3.7	89.7 ± 0.5	/	2.2	+ 23.01 ± 5.09	[155]
CS	CS-g-N-Phe_20.2%_/insulin PECs/SDS	130.7 ± 4.0	93.7 ± 2.4	/	5.8	− 36.52 ± 5.91	[155]
CS	PC6/CS NPs	/	/	/	16.2	/	[156]
CS	insulin-loaded chitosan-DET nanoparticles	200	/	6.4	/	+ 17	[157]
CS	insulin/CS-PLGA NPs	150	40.61	8.41	/	/	[158]
CS/VitB12^e)^	VitB12-Chi-CPNPs	250	/	7.83 ± 0.46	26	+ 32.56 ± 2.34	[159]
156CS/HA	CNPs	158.6 ± 2.4	/	28.8	13.8	− 21.3 ± 0.7	[160]
CS/ALG	CDA NPs	143.3 ± 10.8	61.14 ± 1.67	3.36 ± 0.09	15	19.5 ± 1.6	[161]
CS/ALG/Casein	NiM	1106 ± 232.5	51.1 ± 3.3	/	9.57–10.36	− 19.6 ± 5.7	[162]
HA^f^	NP_HA-SH_	593 or 759	91.1	38	11.3	− 7 or − 5	[163]
Dextran	P-I-GMs	270	74.1	/	9–10	/	[164]
Dextran	DEX25-PLGA48	342.3 ± 2.45	92.46 ± 1.90	27.76 ± 0.57	9.77	/	[81]
Z^g^	I-NP	277 ± 14	77.3 ± 3	76.1 ± 2	4.2	− 39.4 ± 0.2	[165]
Z	I-NP-PEG	263 ± 19	84.8 ± 3	/	10.2	− 38.9 ± 2.3	[165]
Synthetic porous polymers nanocarriers							
Silica	MSN-S@DLPC	3059 ± 1781	/	12.52 ± 4.36	/	10.1 ± 0.24	[166]
Silica	MSN-NH2	224.8 ± 4.9	/	24.75 ± 3.37	/	14.5 ± 0.75	[166]
Silica	MSN-DC@SB12	2029 ± 267	/	22.18 ± 4.50	/	5.9 ± 0.39	[166]
MOFs	Ins-GOx/ZIF-8	427.5	80.6	9.1	/	45.1	[167]
MOFs	Ins@ZIF-8	125	/	20.2	/	3.18 ± 0.39	[168]
MOFs	GCI@ZIF-8	150	/	24.8	/	7.18 ± 0.37	[168]

^a^Encapsulation efficiency

^b^Loading capacity

^C^Insulin

^d^Alginate

^e^Vitamine B12

^f^Hyaluronic-acid

^g^Zein

## Conclusions and prospects

PNPs have significant potential for oral insulin administration. In recent years, many nanoparticle systems for oral insulin administration have been developed, including natural polymer nanocarriers and synthetic porous polymer nanocarriers. While some are promising, but their long-term efficacy in larger animals and humans must be proven. The majority of the better nanocarrier systems are made from natural small-molecular polymers, but their pore size cannot be controlled. As a result, future studies will focus on manufacturing methods for the synthesis of synthetic porous polymer nanoparticles. Prior to pre-clinical developments, oral insulin products have to be optimized in terms of encapsulation efficiency, loading capacity, gastrointestinal stability, bioavailability and bio-safety and intestinal permeation ability. However, no research has presently demonstrated that these barriers can be fully overcome and entered into clinical trials. This means that the clinical use of oral insulin is still a long way off.

In this review, we describe the controlled release techniques for oral insulin administration and explore the current oral insulin delivery applications of PNPs nanocarriers. Current PNPs production methods face various hurdles, including enhanced bioavailability and the biological stability of insulin in the GI tract. Few researchers have examined the bioavailability of MOFs for oral insulin administration among PNPs. As a result, future studies should explore the applicability of MOFs in humans by determining improvements in their bioavailability. MOF is a unique class of PNP, having porous materials with distinct properties and therefore being considered potential candidates for the construction of oral insulin delivery systems. COFs have a very good drug loading capacity and high drug delivery efficiency due to its unique properties, such as a wide surface area, easy crystallization, a porous structure, and outstanding biocompatibility. COFs as oral insulin carriers are currently being studied and have shown high loading capacity and bioavailability. We may start with the management of pore size design and interlayer space in COFs, then manufacture and precisely load 2.5–3.0 nm of insulin molecules in the pores or between layers to optimise oral insulin bioavailability in the future. We can begin by managing pore size and interlayer space in COFs, then produce and precisely load 2.5–3.0 nm of insulin molecules in the pores or between layers in the future to optimize the oral insulin delivery systems.

## Data Availability

Not applicable.
